# Accelerated two-dimensional cine DENSE cardiovascular magnetic resonance using compressed sensing and parallel imaging

**DOI:** 10.1186/s12968-016-0253-2

**Published:** 2016-06-14

**Authors:** Xiao Chen, Yang Yang, Xiaoying Cai, Daniel A. Auger, Craig H. Meyer, Michael Salerno, Frederick H. Epstein

**Affiliations:** Medical Imaging Technologies, Siemens Medical Solutions, USA Inc., 755 College Rd E., Princeton, NJ 08540 USA; Department of Biomedical Engineering, University of Virginia, Charlottesville, VA 22908 USA; Department of Radiology , University of Virginia, Charlottesville, VA 22908 USA; Department of Cardiology, University of Virginia, Charlottesville, VA 22908 USA

**Keywords:** Cine DENSE, Compressed sensing, Parallel imaging, Myocardial strain, Myocardial tagging, Cardiovascular magnetic resonance

## Abstract

**Background:**

Cine Displacement Encoding with Stimulated Echoes (DENSE) provides accurate quantitative imaging of cardiac mechanics with rapid displacement and strain analysis; however, image acquisition times are relatively long. Compressed sensing (CS) with parallel imaging (PI) can generally provide high-quality images recovered from data sampled below the Nyquist rate. The purposes of the present study were to develop CS-PI-accelerated acquisition and reconstruction methods for cine DENSE, to assess their accuracy for cardiac imaging using retrospective undersampling, and to demonstrate their feasibility for prospectively-accelerated 2D cine DENSE imaging in a single breathhold.

**Methods:**

An accelerated cine DENSE sequence with variable-density spiral k-space sampling and golden angle rotations through time was implemented. A CS method, Block LOw-rank Sparsity with Motion-guidance (BLOSM), was combined with sensitivity encoding (SENSE) for the reconstruction of under-sampled multi-coil spiral data. Seven healthy volunteers and 7 patients underwent 2D cine DENSE imaging with fully-sampled acquisitions (14–26 heartbeats in duration) and with prospectively rate-2 and rate-4 accelerated acquisitions (14 and 8 heartbeats in duration). Retrospectively- and prospectively-accelerated data were reconstructed using BLOSM-SENSE and SENSE. Image quality of retrospectively-undersampled data was quantified using the relative root mean square error (rRMSE). Myocardial displacement and circumferential strain were computed for functional assessment, and linear correlation and Bland-Altman analyses were used to compare accelerated acquisitions to fully-sampled reference datasets.

**Results:**

For retrospectively-undersampled data, BLOSM-SENSE provided similar or lower rRMSE at rate-2 and lower rRMSE at rate-4 acceleration compared to SENSE (*p* < 0.05, ANOVA). Similarly, for retrospective undersampling, BLOSM-SENSE provided similar or better correlation with reference displacement and strain data at rate-2 and better correlation at rate-4 acceleration compared to SENSE. Bland-Altman analyses showed similar or better agreement for displacement and strain data at rate-2 and better agreement at rate-4 using BLOSM-SENSE compared to SENSE for retrospectively-undersampled data. Rate-2 and rate-4 prospectively-accelerated cine DENSE provided good image quality and expected values of displacement and strain.

**Conclusions:**

BLOSM-SENSE-accelerated spiral cine DENSE imaging with 2D displacement encoding can be acquired in a single breathhold of 8–14 heartbeats with high image quality and accurate assessment of myocardial displacement and circumferential strain.

## Background

Imaging myocardial strain is of growing importance for the assessment of heart disease [1–5]. For example, recent studies have shown that strain imaging is effective for quantifying mechanical dyssynchrony and predicting response to cardiac resynchronization therapy in patients with heart failure [6] and that strain imaging can detect subclinical systolic dysfunction in patients with diabetes [7]. Myocardial tagging cardiovascular magnetic resonance (CMR) , a long-established method, has been considered the gold standard method for the noninvasive measurement of myocardial strain [3, 8]. However, recently cine displacement encoding with stimulated echoes (DENSE) [9, 10] has emerged as a strain imaging technique that, compared to tagging, has equivalent accuracy and better interobserver variability [11]. Additionally, strain analysis for cine DENSE is rapid and far less time consuming than for tagging [12–14]. While cine DENSE has advantages in interobserver variability, analysis time, and spatial resolution, it has the disadvantage that data acquisition times are inherently longer than tagging. The longer scan times for cine DENSE occur because DENSE is a phase-contrast method, requiring n + 1 separate acquisitions in order to reconstruct phase images encoded for displacement in n directions [15]. While two-dimensional (2D) grid-tagged images are typically acquired during a clinically-convenient single breathhold [3], common protocols for 2D cine DENSE require two separate breathholds, each acquiring 1D displacement–encoded data and phase-reference data [6] or using a balanced two-point encoding method [15]. Acceleration using data undersampling has the potential to enable cine DENSE scans with 2D displacement encoding in less than 10 s without substantial compromises in spatiotemporal resolution and accuracy, which would represent a clinically-convenient single-breathhold protocol. However, acceleration using conventional parallel imaging (PI) decreases the signal-to-noise ratio (SNR) [16] and may compromise the accuracy of the displacement and strain measurements.

Compressed sensing is a newer technique that is making a major impact on accelerated CMR [17] and which, when combined with PI, may preserve the accuracy of cine DENSE displacement and strain measurements when acceleration is employed. In CS, high-quality images can be recovered from data sampled well below the Nyquist rate provided that the sampling pattern is incoherent, the images are sparse in a transform domain, and a sparsity-promoting iterative reconstruction is used [17]. Cine DENSE imaging may be well-suited for acceleration using CS since the data present spatiotemporal sparsity and there are correlations between data encoded for displacement in different directions. The purposes of the present study were to develop CS-PI-accelerated acquisition and reconstruction methods for cine DENSE, to assess their accuracy for measuring myocardial displacement and strain, and to demonstrate the feasibility of these methods for acquiring high-quality prospectively-accelerated 2D cine DENSE images in a single breathhold.

## Methods

### Pulse sequence and image reconstruction methods

A variable-density spiral cine DENSE sequence was implemented on a 1.5 T scanner (Avanto, Siemens, Erlangen, Germany), where the center of k-space was fully sampled and the outer portion of k-space was undersampled. The undersampled variable-density sequence is a modification of a previously-described spiral cine DENSE sequence [10], which uses phase cycling and through-plane dephasing for artifact suppression [9, 18]. Spiral interleaves were distributed uniformly in k-space within each cardiac phase and were rotated by the golden angle through different cardiac phases to achieve randomness in time. Undersampled k-space data were reconstructed using a modified Block Low-rank Sparsity with Motion guidance (BLOSM) algorithm, a CS method that exploits low-rank spatiotemporal properties within regions or blocks [19]. The BLOSM technique, which was recently developed and applied to accelerate Cartesian first-pass contrast-enhanced perfusion CMR [19], was extended to incorporate sensitivity encoding (SENSE) [20] for the reconstruction of multi-coil data and by using the non-uniform fast Fourier transform (NUFFT) [21] to transform data between the image domain and the spiral trajectories in k-space. In addition, low rank properties in space, time, and through different displacement encoding directions were exploited by applying singular value decomposition (SVD) to 2D matrices containing all these data.

**Fig. 1:**
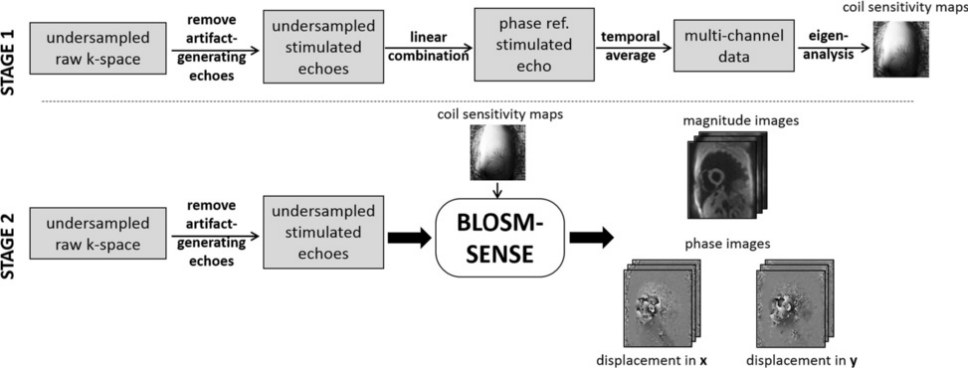
Schematic diagram of the BLOSM-SENSE reconstruction method for 2D cine DENSE. The reconstruction includes (stage 1, *top*) the calculation of sensitivity maps, and (stage 2, *bottom*) the iterative BLOSM-SENSE reconstruction. The undersampled phase-cycled raw k-space data are first subtracted to remove unwanted T1-relaxation echoes. The undersampled isolated stimulated echoes undergo a linear combination to extract the background phase reference stimulated echo. The phase reference data are transformed into images and averaged over time, and eigen-analysis is applied to obtain coil sensitivity maps. At the second stage, the undersampled stimulated echoes isolated from the undersampled raw k-space data are input into the BLOSM-SENSE algorithm. BLOSM-SENSE uses the calculated sensitivity maps to exploit matrix low-rank within regions of SENSE-combined images. The final outputs of the iterative BLOSM-SENSE reconstruction are magnitude and phase images with orthogonal 2D displacement encoding

The first stage of the BLOSM-SENSE method is to calculate sensitivity maps (Fig. 1, *top*). In this step, the phase-cycled raw k-space data undergo subtraction to remove the artifact-generating T1-relaxation echo. Undersampled phase-reference images are either computed directly in the case of simple displacement encoding or using a linear combination in the case of balanced displacement encoding, as previously described [15]. Next, the undersampled phase-reference data are averaged over all cardiac phases*,* from which the coil sensitivity maps are estimated using eigen-analysis [22]. The second stage of reconstruction is to apply the iterative BLOSM algorithm to the SENSE-combined images (Fig. 1, *bottom*). The first frame of the combined images is spatially divided into square regions (blocks) as previously described [19]. The blocks are propagated through the cardiac phases (in this case without motion tracking) and the time series of blocks are grouped into clusters which contain structurally-similar and temporally-related contents. Blocks at the same spatiotemporal locations but from different displacement encoding directions are also grouped into the same cluster since the underlying object is the same. Each cluster is rearranged into a 2D Casorati matrix where each block within the cluster becomes a column of the 2D matrix and blocks from different cardiac phases and encoding directions comprise the rows. The Casorati matrix undergoes SVD and the singular values undergo soft thresholding to promote low rank, as the smaller singular values mainly represent noise and incoherent artifact. The denoised clusters are calculated using the thresholded singular values and are merged back into complete images. Data fidelity is calculated for each coil. The algorithm proceeds for a predefined number of iterations; in this case 200 iterations were empirically chosen. The final outputs are complex-valued images encoded for orthogonal 2D displacement. When acquired, fully-sampled datasets were reconstructed using the sum-of-squares (SOS) method. Specifically, the calculation to obtain the magnitude and phase-valued images followed the methods in reference [10], where the magnitude takes the SOS of the absolute value of all the channels and the phase is calculated using the sum of the phase images of all the channels. To show the artifact level due to undersampling without a CS reconstruction, undersampled datasets were also reconstructed using SENSE alone. In addition, to justify the use of BLOSM-SENSE without motion tracking, we also reconstructed all of the in vivo images using BLOSM-SENSE with motion tracking and quantitatively compared the results.

### Computer-generated heart phantom for evaluation of BLOSM-accelerated cine DENSE

**Fig. 2:**
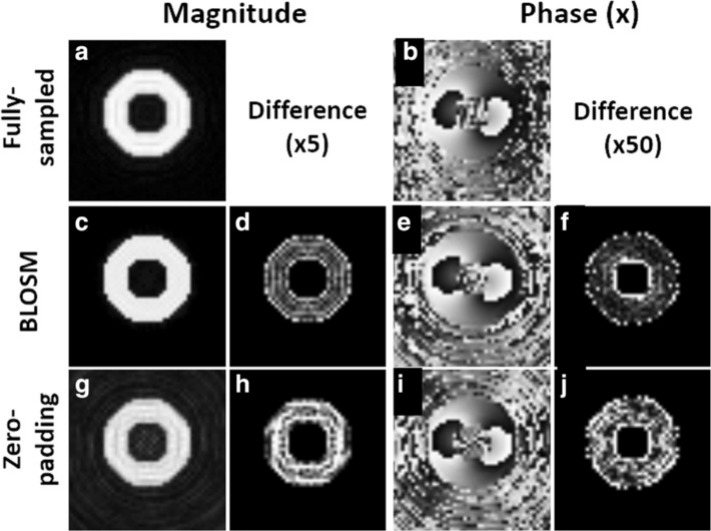
Example reconstructions of a computer simulated cardiac-contraction phantom. Using full data sampling (*top row*) and rate-4 undersampling with BLOSM (*middle row*) and zero-padding (*bottom row*), reconstructed magnitude (**a**,**c**,**d**,**g**,**h**) and phase (**b**,**e**,**f**,**i**,**j**) images with displacement encoding applied in the **x** direction at end-systole are shown. Fully-sampled data reconstructed with NUFFT (**a**,**b**), undersampled data reconstructed using BLOSM (**c**,**e**), and undersampled data reconstructed using zero-padding NUFFT (**g**,**i**) are shown in different rows. Difference images (**d**,**f**,**h**,**j**) computed by subtracting reconstructed images from fully-sampled images are also shown (difference amplified by 5 and 50 times for magnitude and phase images, respectively, for display purposes). BLOSM reduced error as compared to zero padding compared to the fully-sampled images

Computer-simulated data have previously been used to evaluate new algorithms for improving cine DENSE [13, 23]. In the present study, a computer-simulated cardiac phantom was generated to mimic 2D cine DENSE data representing a contracting left ventricle in a short-axis view. As shown in Fig. 2, two concentric circles were generated to represent the epicardial and endocardial borders. Cardiac contraction and relaxation were simulated by deforming the phantom, as described previously [13] . No additional artifact-generating echoes were simulated [24]. Noise was added to the complex-valued images, and fully-sampled k-space data on variable-density spiral trajectories were computed using the NUFFT. Accelerated (undersampled) acquisitions and BLOSM reconstructions were simulated at rate 2, 4 and 8 using the spiral trajectories listed in Table 1. Single-coil acquisitions were assumed for the phantom. The undersampled datasets were also reconstructed using zero padding and NUFFT.

**Table 1:** Parameters for the variable density spiral trajectories for fully-sampled and accelerated acquisitions

	Number of interleaves	Initial sampling density	Ending sampling density
Full	8	3	0.8
Rate 2	4	1.2	0.4
Rate 4	2	1.2	0.12
Rate 8	1	1.2	0.03

The reference sampling density of 1 corresponds to the Nyquist rate

### Volunteer and patient scans

CMR data were collected from seven healthy volunteers (4 male; mean age 29, range 23–33 years) and seven patients (3 male; mean age 64, range 39–79 years) with known or suspected heart disease. The scans were performed in accordance with protocols approved by our institutional review board after informed consent was obtained. Short-axis 2D cine DENSE images of the left ventricle (LV) at a mid-ventricular level were collected on a 1.5 T CMR scanner (Avanto, Siemens, Erlangen, Germany) with a 5-channel body-spine combined RF coil array. For each volunteer scan, fully-sampled cine DENSE datasets with 2D in-plane displacement encoding and 6–8 spiral interleaves per image were acquired within a long breathhold (20 to 26 heartbeats). For each patient scan, two fully-sampled cine DENSE datasets with orthogonal 1D in-plane displacement encoding and 6 spiral interleaves per image were acquired within a shorter breathhold (14 heartbeats). Other sequence parameters included field of view = 280-340 × 280-340 mm^2^, spatial resolution = 1.8-2.4 × 1.8-2.4 mm^2^, slice thickness = 8 mm, ramped flip angle with the last flip angle = 15°, TR = 9.8 ms, TE = 1.3 ms, and temporal resolution = 19.6 ms. In addition, for both healthy volunteers and patients prospectively undersampled datasets at acceleration rates 2 and 4 (with 4 and 2 spiral interleaves per image, respectively) with 2D displacement encoding were acquired with shorter breathholds of 14 and 8 heartbeats, respectively. With a temporal resolution of 19.6 ms per cardiac phase, all datasets acquired 20–38 cardiac phases (35–38 phases for volunteers, and 20–30 phases for patients). These images covered on average 655 ms, and approximately 75 % of the cardiac cycle. The fully-sampled datasets provided reference images, and retrospective undersampling of these datasets was used to evaluate the new acceleration methodologies. Prospectively acquired undersampled cine DENSE datasets were used to demonstrate true acceleration. Parameters describing the fully-sampled and accelerated spiral trajectories are listed in Table 1.

### Image quality assessments and functional analysis

For retrospectively accelerated data, image quality for the various reconstruction algorithms was quantified using the relative root mean square error (rRMSE). Complex-valued images were used in the rRMSE calculations shown below. rRMSE values were averaged over all the cardiac phases and rRMSE was defined as

1 $$ \mathrm{rRMSE} = \frac{1}{N}\sqrt{{\displaystyle \sum}\frac{{\left|{\boldsymbol{m}}_{\boldsymbol{ref}}-{\boldsymbol{m}}_{\boldsymbol{recon}}\right|}^2}{{\left|{\boldsymbol{m}}_{\boldsymbol{ref}}\right|}^2}} $$

where *N* is the number of pixels, ***m***_***ref***_ are the fully-sampled images and ***m***_***recon***_ are the images reconstructed from retrospectively undersampled data. rRMSE for regions of interest (ROIs) in the phase-reconstructed images were also calculated, as measurement of phase is critical for computation of displacement and strain in DENSE. ROIs were selected that delineated the myocardium.

Myocardial displacement and strain were computed using previously published semi-automatic cine DENSE analysis algorithms [12, 14]. To calculate myocardial displacement and strain, endocardial and epicardial contours were manually delineated at one cardiac phase and propagated automatically to all other cardiac phases [12]. After this segmentation process, the phase-reconstructed images were phase unwrapped and displacement and strain were computed within the segmented myocardium [14]. Circumferential strain (E_cc_), the most commonly-reported and accurately-measured strain element [8], was selected in this study to evaluate the reconstruction algorithms. Short-axis images were segmented into 6 regions according to the standard American Heart Association model of the left ventricle [25] and segmental E_cc_ values at all cardiac phases were recorded. Linear correlation and Bland-Altman analyses were performed to assess the relationship between the accelerated reconstructions and the reference data at each spatiotemporal E_cc_ data point (1488 points in total for 7 volunteers, 1062 points in total for 7 patients). When comparing E_cc_ from retrospectively-undersampled images to fully-sampled images, only the volunteer data were used because these fully-sampled DENSE images with 2D displacement encoding were acquired in a single breathhold. All of the rRMSE and Bland-Altman plots included all of the data from all acquired cardiac phases.

To show the behavior of BLOSM-SENSE compared to SENSE under the condition of low SNR, for one dataset various levels of noise were added to the fully-sampled data to simulate different SNR values (-10 dB to -2 dB), and the data were then rate-4 undersampled and reconstructed using both reconstruction methods.

## Results

### Computer-generated heart phantom for evaluation of BLOSM-accelerated cine DENSE

**Fig. 3:**
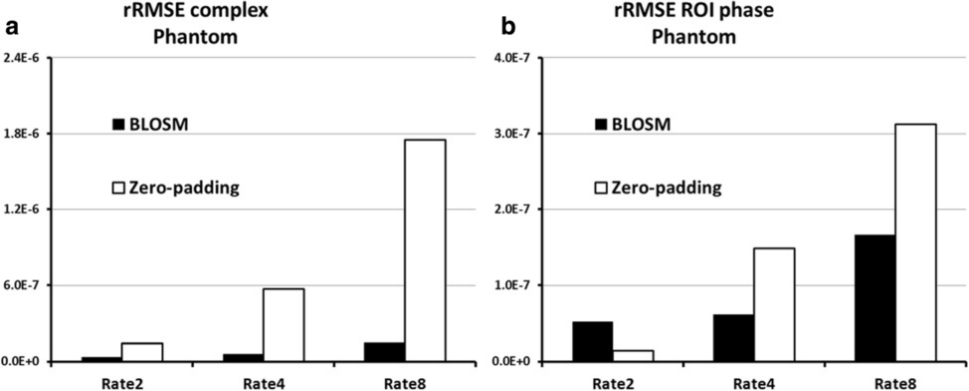
rRMSE of images from a computer-simulated cardiac-contraction phantom at different accelerate rates. rRMSE of the complex-valued images is shown in (**a**) Regional rRMSE over the myocardium of the phase images was calculated and shown in (**b**) At the lower acceleration rate of 2, BLOSM and zero-padding NUFFT performed similarly. At higher acceleration rates of 4 and 8, BLOSM had lower rRMSE

**Fig. 4:**
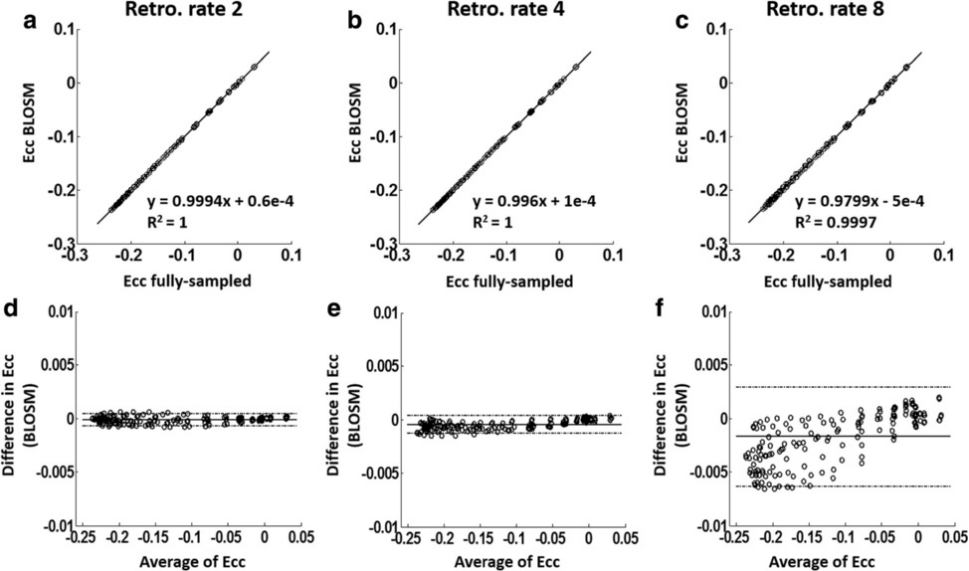
E_cc_ linear correlation and Bland-Altman analysis for a computer-simulated cardiac-contraction phantom. Linear correlation (**a**-**c**) and Bland-Altman analysis (**d**-**f**) of E_cc_ calculated using BLOSM with rate-2, rate-4 and rate-8 undersampling compared to fully-sampled simulations are shown. Excellent agreement was achieved at all acceleration rates. Note that the y-axis range on the Bland-Altman graphs are much smaller than the E_cc_ range

Figure 2 shows example images from a computer-generated heart phantom that simulate rate-4 acceleration. Simulated magnitude and phase images (with displacement encoding applied in the **x** direction) at end-systole are shown. Difference magnitude and phase images of the phantom computed by subtracting the reconstructed images from the fully-sampled images are also shown, demonstrating artifact reduction using BLOSM compared to zero-padding. rRMSE increased a small amount from rate-2 to rate-4 acceleration, and increased more between rate-4 and rate-8 acceleration (Fig. 3). Furthermore, as shown in Fig. 4, linear correlation and Bland-Altman analysis of E_cc_ calculated from BLOSM-reconstructed images showed excellent agreement with the fully-sampled images at all acceleration rates, with greater errors as the acceleration rate increased. Note that on the Bland-Altman plot, the difference in E_cc_ is much smaller than the average of E_cc_.

### Retrospective undersampling of fully-sampled volunteer and patient scans

**Fig. 5:**
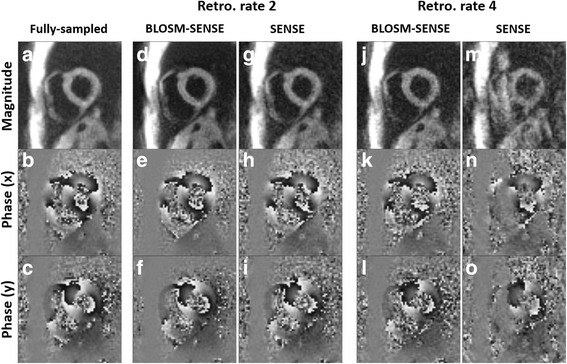
Example reconstructions of retrospectively rate-2 and rate-4 accelerated 2D cine DENSE from a healthy volunteer. Magnitude (*top row*) and phase images (*middle and bottom rows*) with displacement encoding applied in the **x** and **y** directions at end-systole are shown. Fully-sampled data were reconstructed with NUFFT and SOS (**a**-**c**), and rate-2 and rate-4 accelerated data were reconstructed using BLOSM-SENSE (**d**-**f**, **j**-**l**), and SENSE (**g**-**i**, **m**-**o**). BLOSM-SENSE achieved better image quality than SENSE at both acceleration rates

**Fig. 6:**
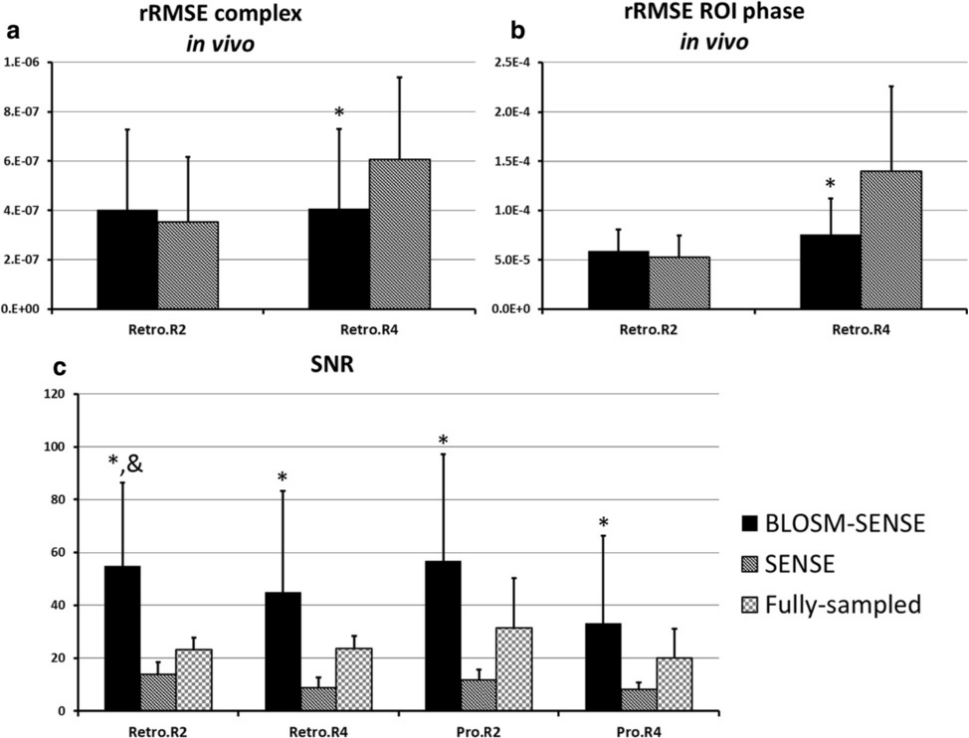
Quantitative analysis of the performance of various reconstruction methods applied to imaging of volunteers and patients. Relative root mean square error (rRMSE) and signal-to-noise ratio (SNR) are shown, with error bars indicating one standard deviation. (**a**) rRMSE of the complex-valued images; (**b**) Regional rRMSE over the myocardium of the phase images; (**c**) SNR values. At an acceleration rate of 2, both methods offered similar rRMSE, although BLOSM-SENSE presented higher SNR. At an acceleration rate of 4, BLOSM-SENSE showed significantly lower rRMSE compared to SENSE for complex and phase data (*p < 0.05, ANOVA). BLOSM-SENSE showed significantly higher SNR compared to SENSE reconstruction (*p < 0.05 v.s. SENSE, &*p* < 0.05 v.s. fully-sampled, ANOVA)

**Fig. 7:**
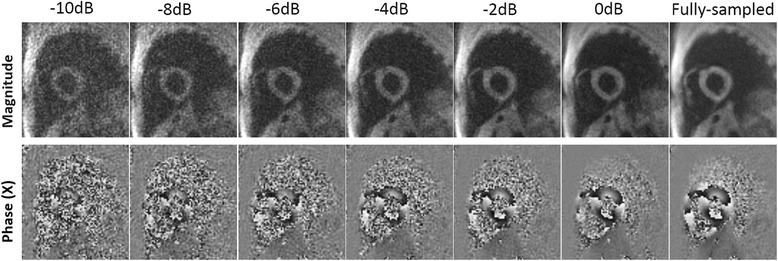
Example magnitude and phase images demonstrating the effects of decreasing SNR on BLOSM-SENSE reconstructions. Simulations were performed by adding noise to fully-sampled reference data to achieve SNR of 0 dB to -10 dB. Retrospectively rate-4 undersampled data were computed and reconstructed using BLOSM-SENSE

**Fig. 8:**
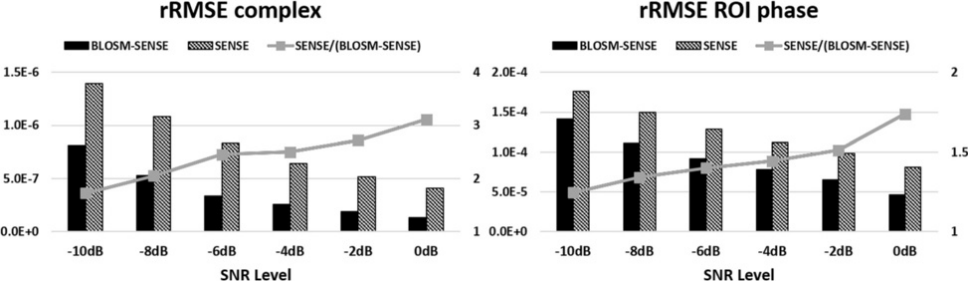
rRMSE analysis of simulated SNR levels for retrospectively rate-4 undersampled data reconstructed using BLOSM-SENSE and SENSE. rRMSE values are shown in bars and the rRMSE ratio for SENSE/BLOSM-SENSE is shown by the gray line (right-side y-axis)

Example magnitude and phase images at end-systole from rate-2 and rate-4 retrospectively-accelerated fully-sampled volunteer scans are shown in Fig. 5 for both BLOSM-SENSE and SENSE reconstructions. Similar image quality was achieved using BLOSM-SENSE compared to the reference at both rate-2 and rate-4 accelerations, with minimal artifacts. Importantly, the phase information in the LV was well maintained. Metrics of image quality quantifying the different reconstruction methods and different acceleration rates for all volunteers and patients are shown in Fig. 6. BLOSM-SENSE generally performed better than SENSE in terms of rRMSE and SNR, particularly at an acceleration rate of 4. The comparison of BLOSM-SENSE without and with motion tracking showed that the resulting images appeared nearly identical, and this qualitative assessment was confirmed by quantitative error metrics. Specifically, for rate-4 acceleration, rRMSE for complex images and phase images reconstructed using BLOSM-SENSE without motion tracking differed by just 4.5 and 2 % compared to BLOSM-SENSE reconstruction with motion tracking. Similarly, for rate-2 acceleration, rRMSE for complex images and phase images reconstructed using BLOSM-SENSE without motion tracking differed by just 7.4 and 4.4 % compared to BLOSM-SENSE reconstruction with motion tracking. For the dataset where various levels of noise were added to simulate different SNR values (-10 dB to -2 dB), example BLOSM-SENSE reconstructed magnitude and phase images are shown in Fig. 7 and the rRMSE analysis for both BLOSM-SENSE and SENSE are shown in Fig. 8. Overall, BLOSM-SENSE provided lower rRMSE than SENSE at all SNR levels. When SNR was lower than -6 dB, the advantage of BLOSM-SENSE over SENSE started to decrease, as indicated by the decrease in the rRMSE ratio (gray line).

**Fig. 9:**
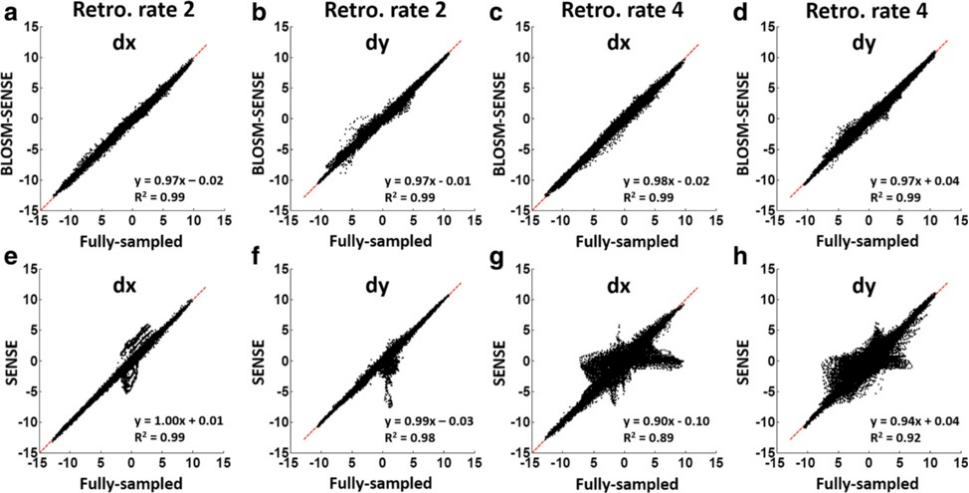
Linear correlation analyses for measurement of myocardial displacement in volunteers and patients. 2D displacements (d**x** and d**y**) calculated from BLOSM-SENSE (**a**-**d**) and SENSE (**e**-**h**) were compared to those from fully-sampled reference data. Results from both retrospectively rate-2 and rate-4 are shown. Good correlations for BLOSM-SENSE are observed at both acceleration rates. SENSE provides moderate correlations at rate 2 and poor correlations at rate 4

**Fig. 10:**
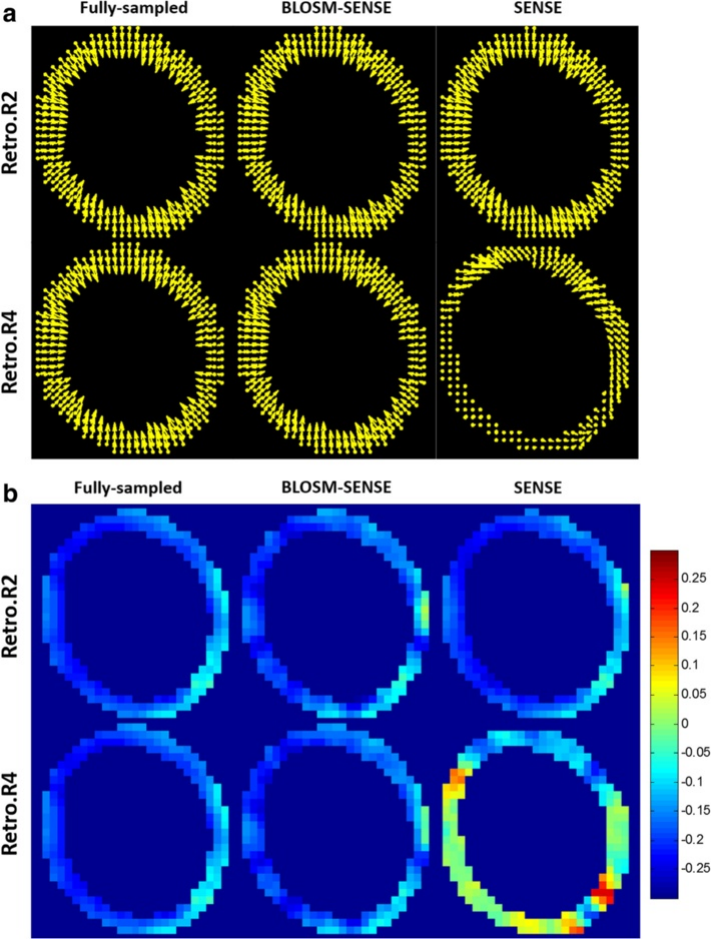
Example displacement and E_cc_ maps at end-systole from one volunteer. Displacement maps (**a**) and E_cc_ maps (**b**) calculated from different methods (columns) and retrospective acceleration rates (rows) are shown. At rate-2, both methods present similar values and patterns to the fully-sampled reference. At rate-4, only BLOSM-SENSE provided accurate displacement and strain assessments

**Fig. 11:**
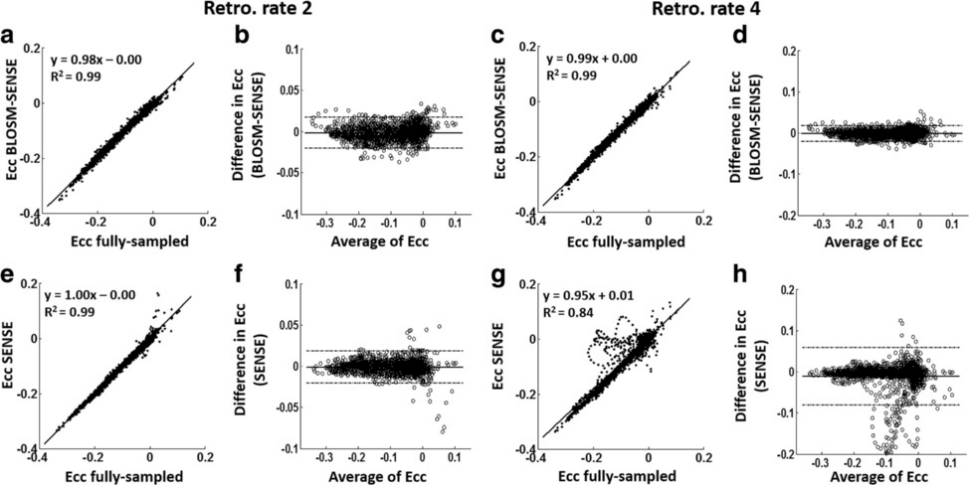
Linear correlation and Bland-Altman analysis of E_cc_ for volunteer imaging with retrospective undersampling. E_cc_ calculated using BLOSM-SENSE (**a**-**d**) and SENSE (**e**-**h**) from retrospectively rate-2 and rate-4 are compared to fully-sampled data. Good correlations and agreements were achieved at both acceleration rates for BLOSM-SENSE compared to SENSE

**Fig. 12:**
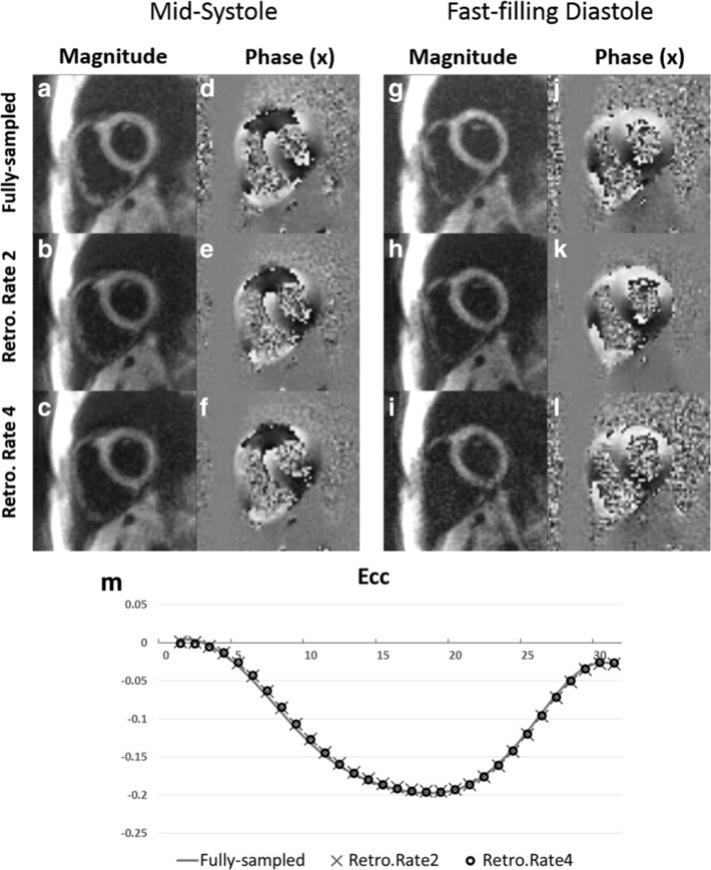
Example BLOSM-SENSE results at rapidly changing cardiac phases. BLOSM-SENSE reconstructions from retrospectively accelerated rate-2 (**b**, **e**, **h**, **k**) and rate-4 (**c**, **f**, **i**, **l**), and fully-sampled reconstructions (**a**, **d**, **g**, **j**) are shown. Global E_cc_-time curves (**m**) are shown for the BLOSM-SENSE and fully-sampled reconstructions. BLOSM-SENSE reconstructions do not show compromised image quality or strain during highly dynamic cardiac phases such as mid systole and early diastole

Figure 9 shows the linear correlation analyses for displacement calculated using BLOSM-SENSE and SENSE for all volunteers and patients at different acceleration rates compared to the fully-sampled data. Displacement values estimated using BLOSM-SENSE showed good correlations for both **x** and **y** encoding directions at rates 2 and 4 acceleration. In contrast, discrepancies are seen for SENSE reconstructions at acceleration rates of both 4 and 2. Figure 10 shows example *in vivo* 2D displacement and E_cc_ maps at end systole for a volunteer computed from the fully-sampled reference images and the retrospectively rate-2 and rate-4 undersampled BLOSM-SENSE and SENSE reconstructed images. Similar values can be observed from the BLOSM-SENSE reconstructions compared to the reference data, however errors are observed using SENSE reconstructions at rate 4. Furthermore, Fig. 11 shows linear correlations and Bland-Altman analyses of E_cc_ for the volunteer data calculated using BLOSM-SENSE and SENSE at different acceleration rates compared to the fully-sampled data. Lastly, retrospectively rate-2 and rate-4 accelerated images from a fully-sampled mid-systolic phase and a fully-sampled fast-filling diastolic phase are shown in Fig. 12, and do not appear compromised by BLOSM-SENSE. Similarly, strain-time curves from these reconstructions (Fig. 12(m)) do not show diminished strain rates during mid systole or early diastole, rapidly changing parts of the cardiac cycle.

### Evaluation of prospectively-accelerated volunteer and patient scans

**Fig. 13:**
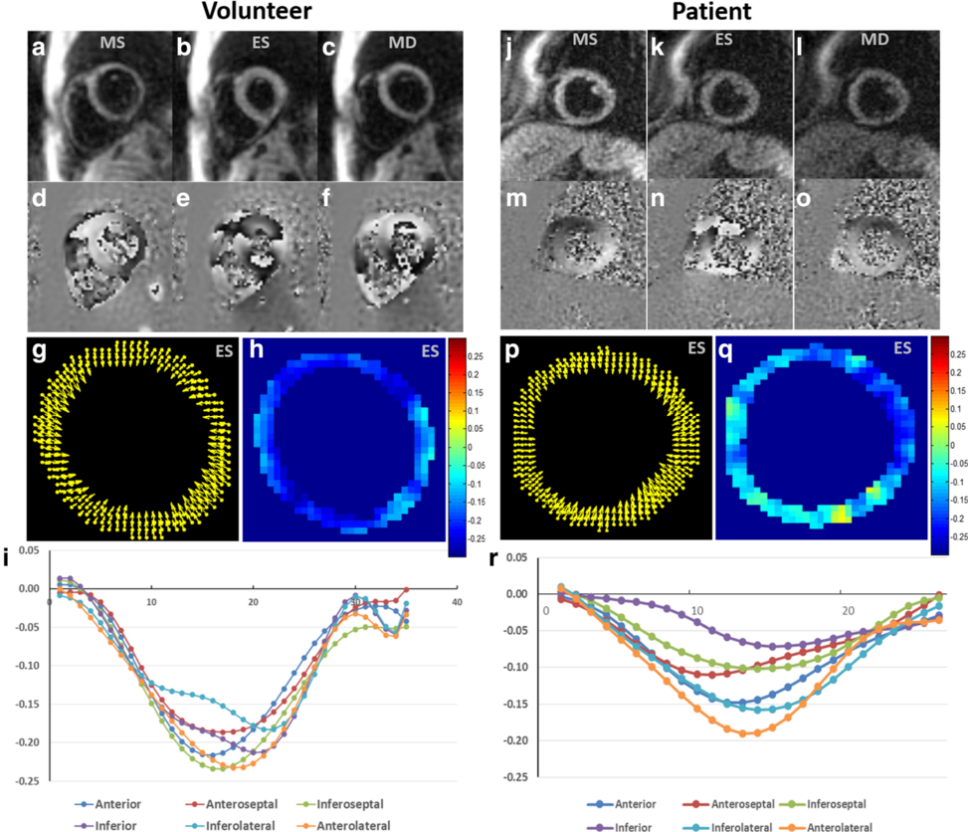
Example prospectively-accelerated BLOSM-SENSE results from rate-4 undersampled data acquired in 8 heartbeats from a volunteer (**a**-**i**) and a patient (**j**-**r**). BLOSM-SENSE reconstructed images, a displacement map, an E_cc_ map and segmental E_cc_-time curves are shown for the volunteer and the patient. Images are shown at mid systole (MS), end systole (ES), and mid diastole (MD). Displacement and E_cc_ maps represent end systole (**g**, **h**, **p**, **q**). With data collected in 8 heartbeats, the images reconstructed by BLOSM-SENSE present high quality, comparable to full sampling. Displacement and strain analyses show typical systolic myocardial contraction patterns and values for the volunteer, and impaired E_cc_ in the septum and inferior wall in the patient

**Fig. 14:**
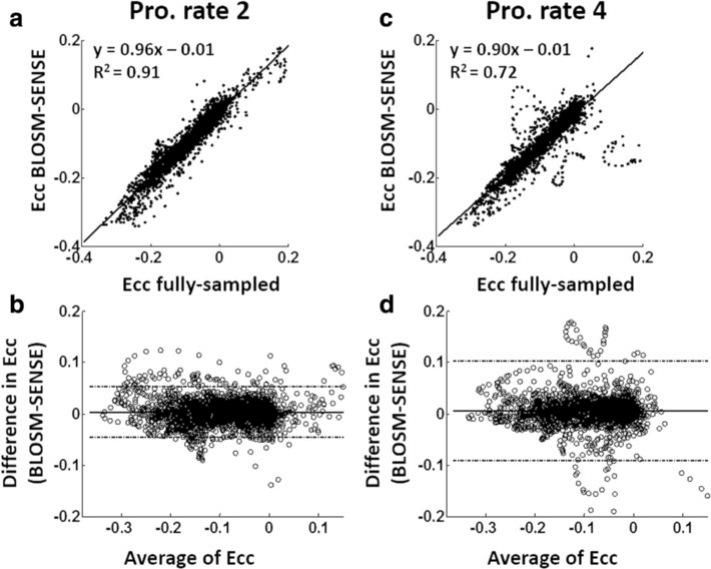
Linear correlation and Bland-Altman analysis of E_cc_ for volunteer and patient imaging using prospective acceleration. E_cc_ calculated using BLOSM-SENSE from prospectively rate-2 (**a**,**b**) and rate-4 (**c**,**d**) accelerated acquisitions are compared to fully-sampled data acquired (in separate breathholds) at matched slice locations. Good agreement was achieved, given the variability incurred by using separate scans acquired during different breathholds

Figure 13 shows examples of rate-4 prospectively-accelerated cine DENSE images for a healthy volunteer and a patient at different cardiac phases (mid systole, end systole, and mid diastole), acquired in breathholds of 8 heartbeats and reconstructed with BLOSM-SENSE. Also shown are representative displacement and E_cc_ maps as well as E_cc_–time curves. Linear correlation and Bland-Altman analyses of these data compared to fully-sampled cine DENSE images acquired separately at matched slice locations show good agreement (Fig. 14), given the variability incurred by using separate scans acquired during different breathholds. The average time for a BLOSM-SENSE reconstruction was 42 min for a common 2D cine DENSE dataset (image size: 128 × 128, 8 k-space spiral interleaves, 5 channels, 2D displacement encoding, 32 cardiac phases) with 200 iterations using MATLAB on a desktop computer (3.4GHz Intel(R)i7 CPU with 12GB RAM).

## Discussion

In this study we developed an accelerated 2D cine DENSE method that can provide accurate 2D displacement and strain maps within a single breathhold of 8-14 heartbeats utilizing variable-density undersampled spiral k-space trajectories for data sampling and BLOSM-SENSE for image reconstruction. Studies using fully-sampled computer-generated phantom data and *in vivo* data with retrospective undersampling demonstrated low rRMSE of CS-PI-reconstructed images and accurate displacement and strain values compared to fully-sampled data. Prospectively-accelerated *in vivo* scans of healthy volunteers and heart disease patients at acceleration rates of 2 and 4 demonstrated good image quality, typical values of displacement and strain for healthy volunteers, and impaired strain for patients. These results demonstrate that undersampled variable-density spiral imaging with BLOSM-SENSE reconstruction can substantially reduce the scan time for cine DENSE CMR compared to conventional protocols while maintaining accurate measurements of displacement and strain.

For CS-PI reconstruction we used a method that exploits the low-rank property in space, time, and among displacement encodings in sub-regions of the images. The BLOSM method has been extended in this study to incorporate PI and exploit data sparsity among RF coils. BLOSM was also extended to handle non-Cartesian k-space data. The properties of BLOSM and its successful application to cine DENSE imaging of the heart suggest that it can be readily extended to tagging and phase-contrast methods that also probe myocardial motion. Regional low-rank methods have recently been used successfully for other applications such as dynamic contrast-enhanced CMR [19, 26], myocardial parameter mapping [27] and imaging of cardiac morphology and function [28]. Other sparsity promoting reconstructions such as x-f methods [29] or others [30] may also be applicable to cine DENSE. A comparison of all the various possible sparsifying transforms that may be applicable to accelerating cine DENSE is beyond the scope of the present study.

Using rate-4 acceleration, a common clinical 2D cine DENSE protocol can be reduced from 28 heartbeats (two breathholds of 14 heartbeats each) to 8 heartbeats. For clinical scanning, this is expected to provide a significant improvement in efficiency, better toleration and compliance by patients, and fewer misregistration errors that may occur due to inconsistencies between different breathholds. Compared to the single-breathhold SENSE-accelerated 2D cine DENSE protocol previously developed by Kim et al [16], the present methods provide three-fold better spatial resolution with a similar or shorter scan time.

While BLOSM makes use of and derives benefit from motion estimation and motion compensation for free-breathing single-shot first-pass perfusion CMR [19], motion estimation is not needed for breathhold cine DENSE imaging and the implementation of BLOSM with motion compensation for segmented acquisitions would be complex. We have reconstructed all of the images in the present study using BLOSM-SENSE both without and with motion tracking, and the results are nearly identical in terms of image appearance and quantitative metrics of image quality such as rRMSE for both rate-2 and rate-4 acceleration. Since the results are equivalent and the method is simpler and less computationally expensive without motion tracking, we chose to use the method without motion tracking for this application.

It is well established that the magnitude of the displacement-encoded stimulated echo signal decreases with time across the cardiac cycle due to T1 relaxation. We used a ramped flip angle method to partially compensate for this effect, however we did observe that SNR decreased and that rRMSE increased for cardiac phases later in the cardiac cycle. Using the present methods, we imaged approximately 75 % of the cardiac cycle, but did not reliably capture late diastole. Further optimization of the ramped flip angle method and potentially using a magnetic field strength of 3 T as recently shown [31] may enable BLOSM-SENSE accelerated cine DENSE imaging of the entire cardiac cycle.

To make fair comparisons in the present study, we held parameters such as readout duration and TR constant. However, acceleration can enable shorter readout durations and shorter TRs, which in turn can reduce spiral blurring artifacts and provide increased temporal resolution. Reduced readout duration also has important implications regarding better spiral cine DENSE image quality at higher field strengths such as 3 T, where off-resonance effects are greater. Thus, acceleration of cine DENSE may have broad implications for achieving shorter scan times, better temporal resolution, use at 3 T, and subsequently higher SNR and/or spatial resolution. Thus these technical innovations will likely lead to better assessment of myocardial strain for patients with heart disease.

## Conclusions

CS-PI-accelerated spiral cine DENSE imaging with 2D in-plane displacement encoding can be acquired in a single breathhold, as short as 8 heartbeats, with high image quality and accurate functional assessments. These methods promise to improve clinical CMR of myocardial strain.

## Abbreviations

BLOSM, block low-rank sparsity with motion guidance; CS, compressed sensing; DENSE, displacement encoding with stimulated echoes; E_cc_, circumferential strain; HARP, harmonic phase; NUFFT, non-uniform fast Fourier transform; PI, parallel imaging; ROI, region of interest; rRMSE, relative root mean square error; SENSE, sensitivity encoding; SOS, sum-of-squares; SVD, singular value decomposition.
